# Evolutionary changes of the novel Influenza D virus hemagglutinin-esterase fusion gene revealed by the codon usage pattern

**DOI:** 10.1080/21505594.2018.1551708

**Published:** 2018-11-26

**Authors:** Ziqing Yan, Ruyi Wang, Letian Zhang, Binger Shen, Ningning Wang, Qiuhua Xu, Wei He, Wanting He, Gairu Li, Shuo Su

**Affiliations:** MOE Joint International Research Laboratory of Animal Health and Food Safety, Jiangsu Engineering Laboratory of Animal Immunology, College of Veterinary Medicine, Nanjing Agricultural University, Nanjing, China

**Keywords:** IDV, HEF, codon usage, mutation pressure, natural selection, evolution

## Abstract

The codon usage pattern can reveal the adaptive changes that allow virus survival and fitness adaptation to their particular host, as well as the external environment. Although still considered a novel influenza virus, there is an increasing number of influenza D viruses (IDVs) reported. Considering the vital role of the hemagglutinin-esterase fusion (HEF) gene in receptor binding, receptor degradation, and membrane fusion, we investigated the codon usage pattern of the IDV HEF gene to better understand its adaptive changes during evolution. Based on the HEF gene, three groups including, D/OK, D/660, and D/Japan were identified. We found a low codon usage bias, which allowed IDV to replicate in the corresponding hosts by reducing competition during evolution, that was mainly driven by natural selection and mutation pressure, with a profound role of natural selection. Furthermore, the interaction between the codon adaption index (CAI) and the relative codon deoptimization index (RCDI) revealed the adaption of IDV to multiple hosts, especially cattle which is currently considered its reservoir. Additionally, similarity index (SiD) analysis revealed that the swine exerted a stronger evolutionary pressure on IDV than cattle, though cattle is considered the primary reservoir. In addition, the conserved PB1 gene showed a similar pattern of codon usage compared to HEF. Therefore, we hypothesized that IDV has a preference to maintain infection in multiple hosts. The study aids the understanding of the evolutionary changes of IDV, which could assist this novel virus prevention and control.

## Introduction

As a novel genus of the Orthomyxoviridae, influenza D virus (IDV) was first identified in 2011 and provisionally named C/swine/Oklahoma/1334/2011 (C/OK) [1]. Although IDV was initially detected in swine, it was considered to be an important causative agent of bovine respiratory disease, with cattle as the primary reservoir [2]. Additionally, serological surveys revealed that IDV infects small ruminants [3], as well as ferrets, which are the preferred animal model to study influenza virus human infections [2]. Moreover, with the increasing number of cases reported in many countries [4–8], it is urgent to research the adaption of this multi-host influenza virus during its evolution.

Generally, the redundancy of the genetic code allows individual amino acids to be translated by more than one codon, and thus codons encoding the same amino acid are referred to as synonymous codons [9]. However, synonymous codons are not randomly selected, a phenomenon known as codon usage bias [10,11]. This phenomenon allows viruses to efficiently survive and adapt to their corresponding hosts, as well as the environment [12,13]. The codon usage pattern is influenced by natural or translational selection and mutation pressure [14,15], as well as other factors such as, replication, selective transcription protein structure, protein hydrophobicity and hydrophilicity, and the external environment [12,16,17]. Most RNA viruses have a low codon usage bias [12,13,18,19], which allows efficient replication in the host cell by lowering the competition with the host genes. Comparing the codon usage pattern of virus to their specific hosts helps us better understand the fitness and escape adaptations that take place during virus evolution [20]. Furthermore, influenza viruses characterize by a complete dependence on the host during replication and, in addition, their codon usage pattern is adapted to their particular hosts [21].

Similar to influenza C virus (ICV), the IDV genome consists of seven RNA segments. Interestingly, the hemagglutinin-esterase fusion glycoprotein (HEF) has the same functions as the hemagglutinin (HA) and neuraminidase (NA) proteins of influenza A virus (IAV) and influenza B virus (IBV) [22]. The HEF is crucial to receptor binding, damaging, and membrane fusion [1]. Furthermore, except for the HEF gene and the conserved PB1 gene, the other internal genes are frequently associated with reassortment [23]. Here, we analyzed all publicly available IDV HEF and PB1 gene sequences in terms of codon usage patterns. Detailed genetic analyses of emerging IDV are important for understanding and estimating the risk of ongoing transmission amongst mammals and potential public health risks as well as for developing effective countermeasures.

## Materials and methods

### Sequence data and phylogenetic analysis

A total of 38 complete coding sequences of the IDV HEF and 27 of the PB1 genes were downloaded from GenBank of National Center for Biotechnology Information (https://www.ncbi.nlm.nih.gov/genbank/). After removal a low-quality sequence, D/bovine/France/2986/2012, 37 sequences of HEF were left for analysis. The detailed informations of the sequences, including accession number, country and year of isolation, are listed in supplementary materials (Table S1). Sequences were aligned by muscle in MEGA 7.0 [24]. The neighbor joining tree was reconstructed based on a p-distance substitution model implemented in MEGA 7.0 [24] with the bootstrap value set at 1,000.

### Codon usage bias parameters

#### Nucleotide content

The content of each nucleotide (A%, U%, G%, C%), AU, and GC were calculated using BioEdit. In addition, the nucleotide frequencies of synonymous codons at the third position (A_3_%, U_3_%, G_3_%, C_3_%) were calculated using CodonW (v1.4.2). The frequencies of synonymous G + C at the first (GC_1_), second (GC_2_), and third codon positions (GC_3_) were calculated using the online website: cusp (http://www.bioinformatics.nl/emboss-explorer/). The G + C at the first and second positions (GC_12_) was also calculated. The codons AUG, UGG, and the termination codons (UAA, UGA, UAG) were removed from the analysis.

#### Relative synonymous codon usage (RSCU)

To find the most commonly used synonymous codons, the RSCU values for 59 codons were calculated using MEGA7.0. A RSCU value of 1 indicates that the codons are used equally [25]. Codons with RSCU values < 0.6 > 1.6 represent “under-represented” and “over-represented” codons, respectively [26].

#### Principal component analysis (PCA)

PCA, a multivariate statistical method [27], analyses the major tendency of codon usage patterns. To reduce the misleading that amino acid composition exerts on the codon usage, each strain is represented as a 59-dimensional vector, with each dimension corresponding to the RSCU value for each sense codon [28].

#### Effective number of codons (ENC)

The ENC is a useful tool to evaluate the degree of codon usage bias. The ENC value ranges from 20 (only one codon was used) to 61 (all synonymous codons were used equally) [29]. The smaller the value, the stronger the codon preference is. A value less than 35 is indicative of a strong preference [30]. The value is calculated as follows:

$$ENC=2+\frac{9}{{\begin{array}{c} -\\ F \end{array}}_{2}}+\frac{1}{{\begin{array}{c} -\\ F \end{array}}_{3}}+\frac{5}{{\begin{array}{c} -\\ F \end{array}}_{4}}+\frac{3}{{\begin{array}{c} -\\ F \end{array}}_{6}}$$

The $F_{k}$ (k = 2,3,4,6) represents the mean $F_{k}$ value in the k-fold degenerate amino acid family, and $F_{k}$ was calculated as:

$$F_{k}=\;\frac{nS-1}{n-1}$$

n represents the total number of codons to the corresponding amino acid. Additionally, the S was calculated as follows:

$$S\;=\overset{k}{\underset{i=1}{\sum }}{\left(\frac{n_{i}}{n}\right)}^{2}$$

In the formula, the $n_{i}$ means the total number of the $i_{th}$ codon for the corresponding amino acid.

ENC-plot analysis consists in plotting GC_3s_ in the abscissa and the ENC value in the ordinate and is used to investigate the major factors influencing the codon usage bias, like mutation pressure, natural selection, and nucleotide composition [29]. If mutation pressure is the only factor driving codon usage bias, the points will lie on the standard curve. Alternatively, if the points sit below the standard curve, it is indicative of that except for mutational pressure, other factors affect codon usage bias. The expected ENC was calculated as:

ENC _expected_ = 2 + s + $\frac{29}{s^{2}+{\left(1-s\right)}^{2}}$

where s is the composition of the given $GC_{3}$.

#### Parity rule 2 analysis (PR2)

PR2 is applied to explore the relationship of the four-codon amino acids families, with A_3_/(A_3_+U_3_) plotted against G_3_/(G_3_+C_3_), evaluating the equivalence between mutation pressure and natural selection. A = U and G = C means both the axis values are 0.5 and 0.5, indicating a balance between mutation pressure and natural selection [31,32].

#### Neutrality analysis

To determine the effect of mutation pressure on codon usage bias compared to natural selection, neutrality analysis was used. Using GC_3_ as a horizontal coordinate and GC_12_ as the vertical coordinate, the GC_3_ and GC_12_ contents of HEF genes were plotted and a regression line was calculated. Regression lines that fall near the diagonal (slope = 1.0) indicate weak external selection pressure [33], whereas regression curves deviating from the diagonal indicate a significant influence of natural selection on codon usage bias [34].

### Codon adaptation index (CAI)

To reveal the adaptability of the HEF gene to the selected hosts, the CAI were calculated using the CAIcal SERVER (http://genomes.urv.cat/CAIcal/RCDI/) [35]. The hosts including, *Sus scrofa*, *Bos taurus*, and Capra hircu*s* based on serological evidence [3]. The RSCU of the host was obtained from the Codon Usage Database (http://www.kazusa.or.jp/codon/) [36]. The CAI value ranges from 0 to 1.0. The higher the CAI value, the better the virus is adapted to its host [37]. Additionally, the significance regarding the respective clades and hosts were tested in variance analysis with double factor analysis without repetition.

### Relative codon deoptimization index (RCDI)

The relative codon deoptimization index values of the HEF gene were calculated using the RCDI/eRCDI SERVER (http://genomes.urv.cat/CAIcal/RCDI/) [38]. A value of 1.0 means that the codon usage is adapted to the host [39], whereas higher than 1.0 indicates deviation from the host. Additionally, the significance regarding the respective clades and hosts were tested in variance analysis with double factor analysis without repetition.

### Similarity index (SiD) analysis

To reveal the effect of the overall codon usage pattern of hosts on the HEF gene of IDV, the SiD was calculated as follows:

$$R\left(A,B\right)=\frac{\sum_{i=1}^{59}a_{i}\;b_{i}}{\sum_{i=1}^{59}a_{i}{\;}^{2}\sum_{i=1}^{59}b_{i}{\;}^{2}}$$

$$D\left(A,B\right)\;=\frac{1-R\left(A,B\right)}{2}$$

where i is defined as the RSCU value in the synonymous codon usage pattern of the HEF gene, thus, representing the RSCU value for the same codon. D (A, B) is the value of the SiD analysis, indicating the potential impact of the global codon usage of the hosts on the different clades of the HEF gene. The values range from 0 to 1.0 [40].

## Results

### Phylogenetic analysis and PCA

**Figure 1.**
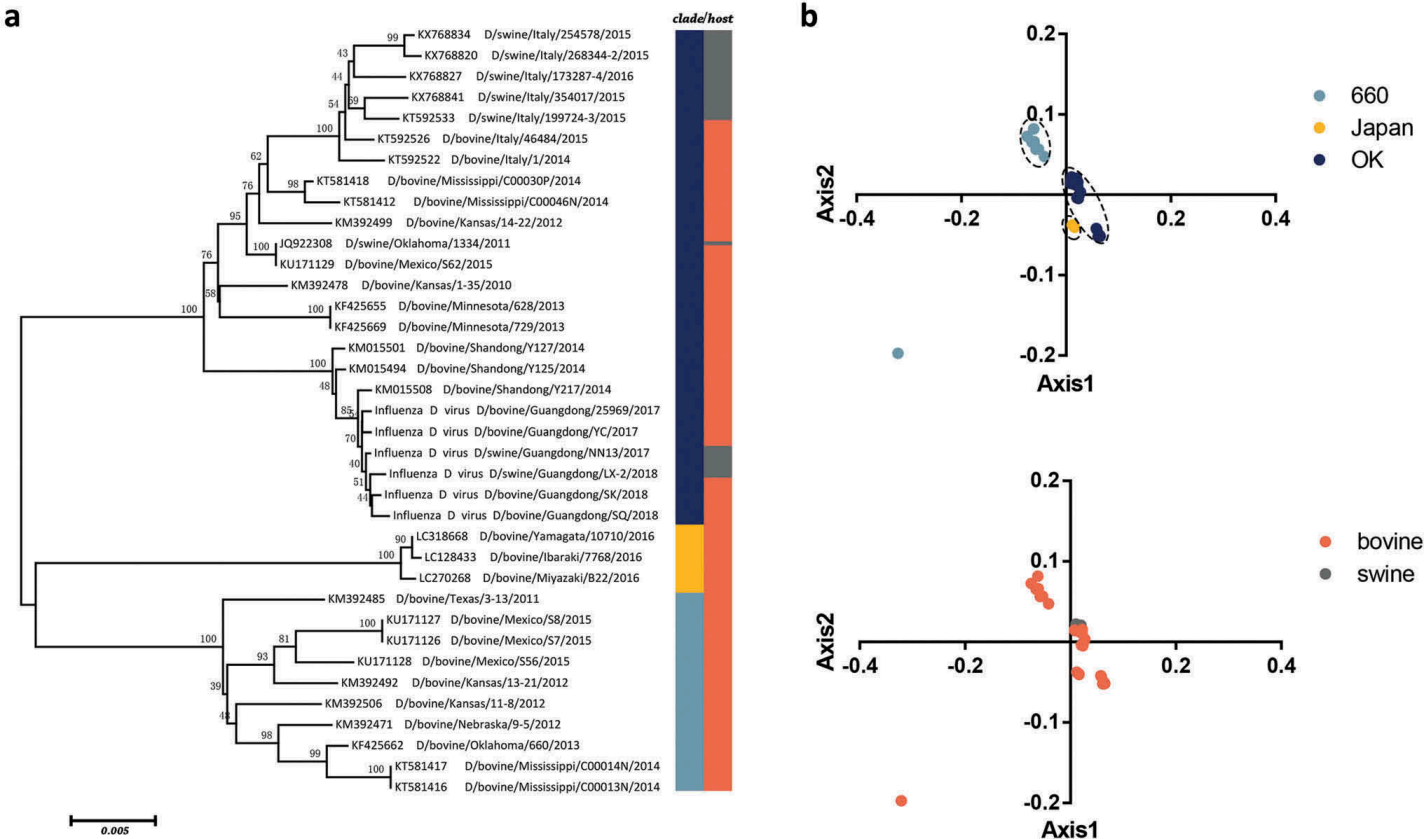
(a) Neighbor joining tree of the IDV HEF gene reconstructed using a p-distance model implemented in MEGA7. (b) PCA of IDV according to different clades and hosts. The D/660, D/Japan and D/OK clades and swine and bovine hosts are represented in light blue, yellow, dark blue, grey, and orange, respectively.

The phylogenetic tree of the HEF gene shows that there are three individual clades: D/OK type, D/660 type, and D/Japan type with high bootstrap values(Figure 1a). This is in agreement with previous studies showing two classical divergent clades. Additionally, the strains discovered in Japan clustered independently from the D/OK and D/660 type [4,41]. The strains from Japan also clustered separately in the PB1 gene phylogeny (Fig S1a).

In PCA analysis, the first and second axis were 37.57% and 23.04%, respectively, accounting for the major source of variation. Then, we explored the distribution of each HEF strain based on the RSCU values on the first two axes (Figure 1b). The HEF strains were mainly divided into three groups: D/660, D/Japan, and the D/OK, except for a D/660 type strain isolated from bovine that clustered separately. It is essential to note that the limited number of sequences might bias the results, and therefore these observations need further confirmation. However, despite the fact that IDV can infect two different hosts, we found several overallaps according to host using PCA analysis indicating that the major codon usage tendency is identical, to some degree, in the two hosts.

### Codon usage bias

#### Nucleotide and synonymous codon composition

We found that the A (proportion: 0.3139 ± 0.00224) and U (proportion: 0.2523 ± 0.001281) nucleotides were used more frequently than C (0.1956 ± 0.00179) and G (0.2380 ± 0.00134). In addition, this tendency was similar for synonymous codons at the third position (Table S1). The average GC_12_ and GC_3_ were 46.79%, and 36.50%, respectively. Furthermore, the more abundant A and U codons were also observed in the PB1 gene (Table S1).

#### Relative synonymous codon usage

In addition, the RSCU value confirmed that U- and A-ended codons are more frequent than C- and G-ended codons for both the HEF or PB1 genes (Table S2). In the HEF gene, among the 18 preferred synonymous codons, 9 ended with A, followed by U-, C- and G-ended codons. When comparing the RSCU values of the individual clades of the HEF gene to the reference hosts, we found that among the 18 frequently used synonymous codons, 17 were consistently used, regardless of clade or host, except for the synonymous codons encoding Tyr. Additionally, the D/660 clade is consistent with bovine while the D/Japan and D/OK clades are consistent with swine (Table 1).

**Table 1.** The RSCU value of 59 codons encoding 18 amino acids according to clades and hosts of HEF gene. The optimal codons are shown in bold.

	Clade			Host			Clade			Host	
Codon	Japan	660	OK	bovine	swine	Codon	Japan	660	OK	bovine	swine
GCU(A)	1.22	1.24	1.17	1.21	1.11	AAU(N)	0.89	0.93	0.84	0.87	0.86
GCC(A)	0.51	0.55	0.52	0.53	0.52	AAC(N)	1.11	1.07	1.16	1.13	1.14
GCA(A)	2.09	2.02	2.11	2.06	2.15	CCU(P)	1.97	1.90	1.92	1.92	1.93
GCG(A)	0.18	0.20	0.21	0.20	0.22	CCC(P)	0.30	0.37	0.32	0.34	0.30
UGU(C)	1.15	1.07	1.09	1.10	1.08	CCA(P)	1.06	1.30	1.31	1.26	1.32
UGC(C)	0.85	0.93	0.91	0.90	0.92	CCG(P)	0.66	0.43	0.46	0.48	0.46
GAU(D)	1.02	1.02	1.06	1.05	1.04	CAA(Q)	1.39	1.50	1.39	1.42	1.42
GAC(D)	0.98	0.98	0.94	0.95	0.96	CAG(Q)	0.61	0.50	0.61	0.58	0.58
GAA(E)	1.42	1.47	1.44	1.45	1.44	CGU(R)	0.00	0.00	0.00	0.00	0.00
GAG(E)	0.58	0.53	0.56	0.55	0.56	CGC(R)	0.00	0.02	0.00	0.01	0.00
UUU(F)	1.00	0.97	0.97	0.97	0.97	CGA(R)	0.26	0.24	0.24	0.24	0.25
UUC(F)	**1.00**	1.03	1.03	1.03	1.03	CGG(R)	0.51	0.24	0.50	0.40	0.50
GGU(G)	0.51	0.47	0.46	0.48	0.45	AGA(R)	3.69	3.55	3.89	3.77	3.83
GGC(G)	0.44	0.56	0.34	0.43	0.36	AGG(R)	1.54	1.95	1.37	1.58	1.43
GGA(G)	1.88	1.89	2.03	1.95	2.04	UCU(S)	1.21	1.44	1.43	1.39	1.45
GGG(G)	1.17	1.09	1.17	1.15	1.15	UCC(S)	0.64	0.42	0.57	0.53	0.55
CAU(H)	1.50	1.50	1.51	1.50	1.50	UCA(S)	1.58	1.79	1.53	1.63	1.53
CAC(H)	0.50	0.50	0.49	0.50	0.50	UCG(S)	0.61	0.46	0.65	0.58	0.63
AUU(I)	1.19	1.02	1.01	1.04	1.01	AGU(S)	0.71	0.93	0.74	0.79	0.78
AUC(I)	0.49	0.61	0.68	0.63	0.68	AGC(S)	1.25	0.96	1.08	1.07	1.06
AUA(I)	1.33	1.37	1.31	1.33	1.31	ACU(T)	1.32	1.40	1.38	1.38	1.40
AAA(K)	1.19	1.17	1.19	1.19	1.19	ACC(T)	0.63	0.66	0.55	0.60	0.57
AAG(K)	0.81	0.83	0.81	0.81	0.81	ACA(T)	1.85	1.84	1.89	1.87	1.86
UUA(L)	0.34	0.59	0.55	0.52	0.62	ACG(T)	0.19	0.10	0.18	0.15	0.18
UUG(L)	2.15	2.29	2.43	2.35	2.38	GUU(V)	1.24	1.35	1.36	1.32	1.40
CUU(L)	1.35	1.43	1.36	1.39	1.33	GUC(V)	0.50	0.42	0.44	0.45	0.43
CUC(L)	0.72	0.56	0.53	0.56	0.53	GUA(V)	1.10	1.02	1.01	1.01	1.03
CUA(L)	0.76	0.64	0.77	0.72	0.79	GUG(V)	1.17	1.22	1.19	1.22	1.14
CUG(L)	0.68	0.50	0.37	0.46	0.35	UAU(Y)	0.98	1.12	0.94	1.01	0.94
						UAC(Y)	1.02	0.88	1.06	0.99	1.06

The low codon usage bias of IDV HEF gene is dominated by natural selection more than mutation pressure

**Figure 2.**
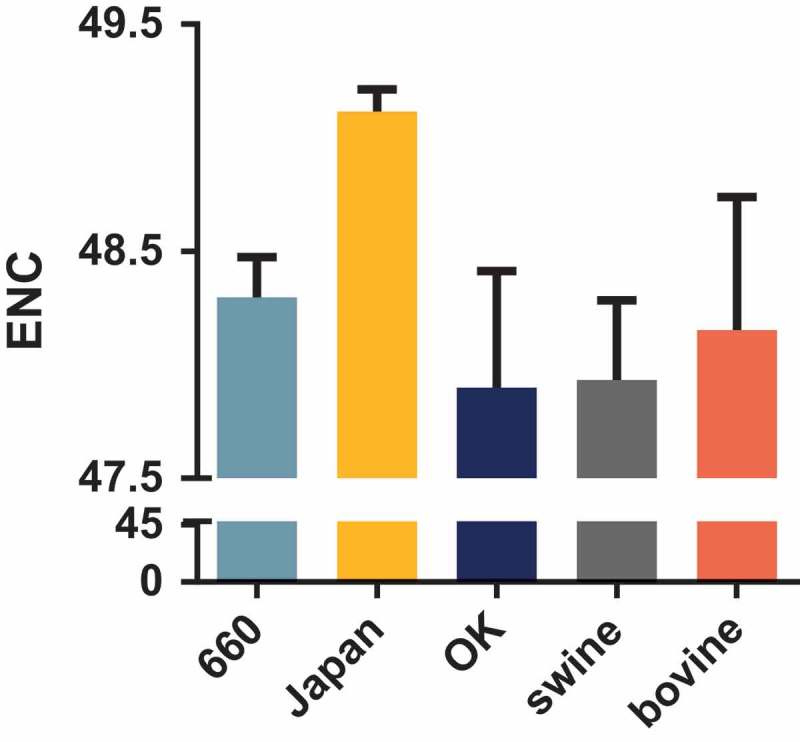
ENC values of the HEF gene of different clades and hosts. The D/660, D/Japan, and D/OK clades and swine and bovine hosts are represented in light blue, yellow, dark blue, grey, and orange, respectively.

**Figure 3.**
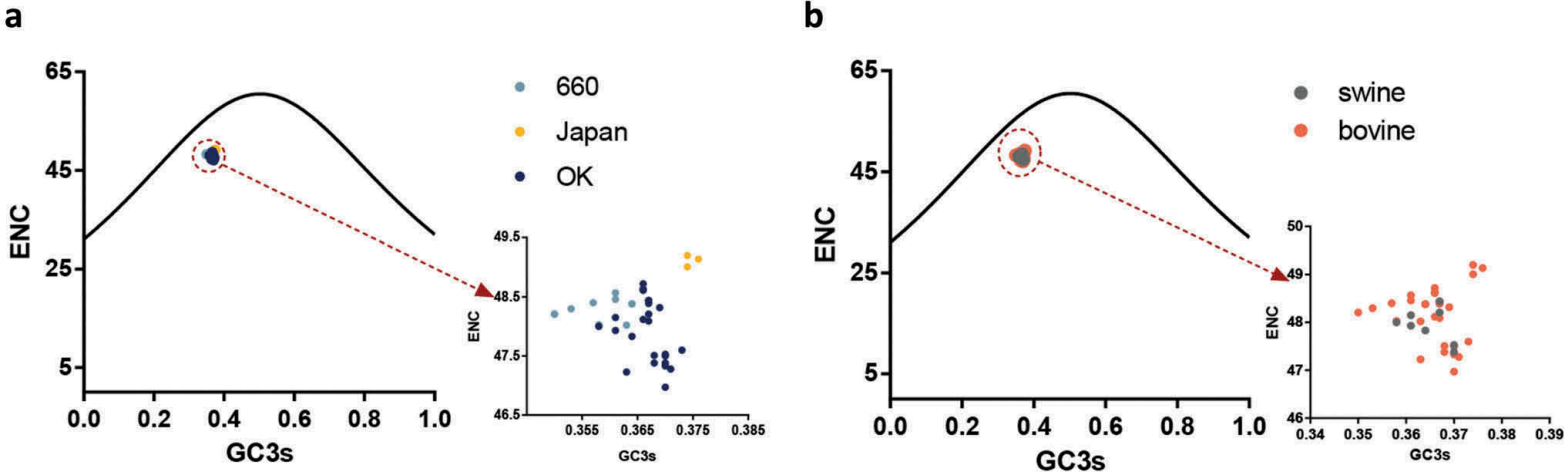
(a, b). ENC-plot analysis of the HEF gene, with ENC against GC_3s_ of different clades and hosts. The black line represents the standard curve when the codon usage bias is determined by the GC_3s_ composition only. The D/660, D/Japan and D/OK clades and swine and bovine hosts are represented in light blue, yellow, dark blue, grey and orange, respectively.

The ENC value revealed that the HEF gene has a low codon usage bias with mean ENC values of 48.3 (± 0.179), 49.12 (± 0.097), 47.90 (± 0.514), 47.93 (± 0.351), and 48.15 (± 0.587) for clades D/660, D/Japan, and D/OK, and the swine and bovine hosts, respectively (Figure 2). Additionally, the mean ENC value of the PB1 gene was 49.40 (± 0.695). Next, we analyzed the factors influencing the codon usage bias of the HEF gene. The ENC-plot analysis according to different clades and hosts (Figure 3a, b) shows that the data points of all the strains are below the standard curve indicative that, except for mutation pressure, other factors like natural selection, drive the codon usage of the HEF gene, in agreement with the PB1 gene (Fig S1B). Additionally, the PR2 plot shows that all the dots are separated from the region (0.5, 0.5), indicative that the degree of mutation pressure and natural selection are not equivalent regardless of clade or host species (Fig S2).

**Figure 4.**
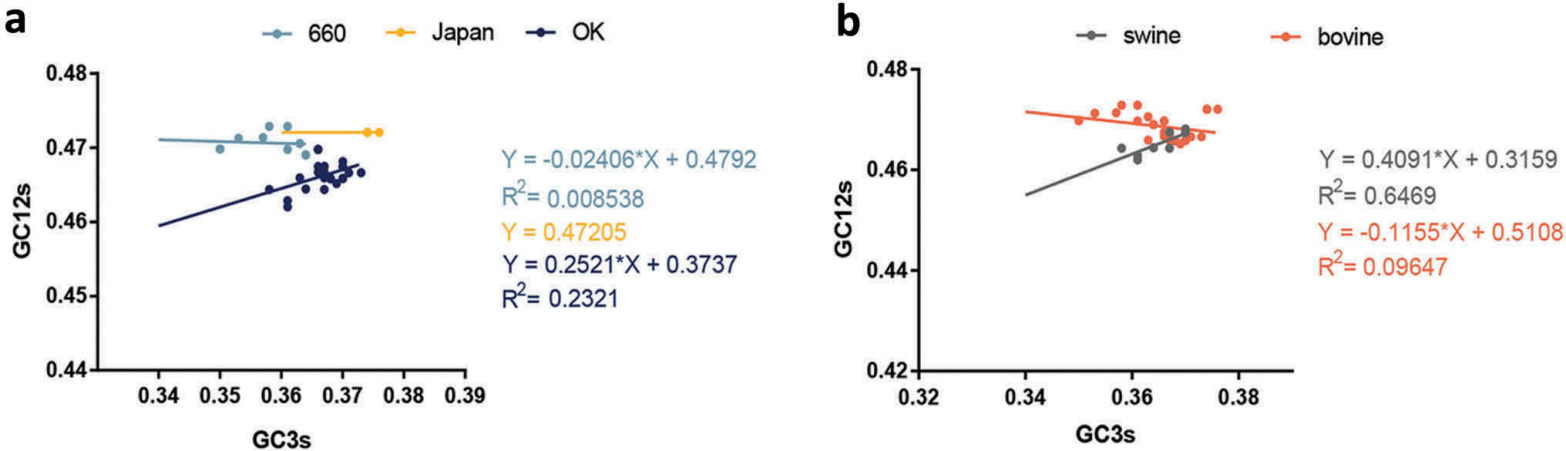
Neutrality plot analysis of GC_3s_ against GC_12s_. a and b are diagrams of different clades and host, respectively. The IDV HEF strains cluster into three clades including: D/660, D/OK and D/Japan, represented in light blue, dark blue, and yellow, respectively. The line and dot of swine and bovine are represented in grey and orange, respectively.

Furthermore, neutrality analysis revealed a narrow distribution and low GC_3_ values (35.04% to 37.58%). In order to decipher the effect of mutational pressure and natural selection in different clades and hosts, regression analysis was performed. We found no significant correlation between GC_12_ and GC_3_ in the D/OK clade (R^2^ = 0.2321, *p* = 0.0171) and in swine (R^2^ = 0.6469, *p* = 0.0161) [12]. In addition, the effect of mutation pressure on the D/660, D/Japan, and D/OK clades and swine and cattle were 2.41%, 0%, 25.21%, 40.91%, and 11.55%, respectively (Figure 4a, b). The above results indicate that natural selection dominates the codon usage over mutation pressure. Additionally, neutrality analysis of the PB1 gene revealed that the effect of mutation pressure was 1.116% with a R^2^ = 0.00361 (Fig S1C).

### IDV displays a complex codon usage adaption and deoptimization pattern in the corresponding hosts

**Figure 5.**
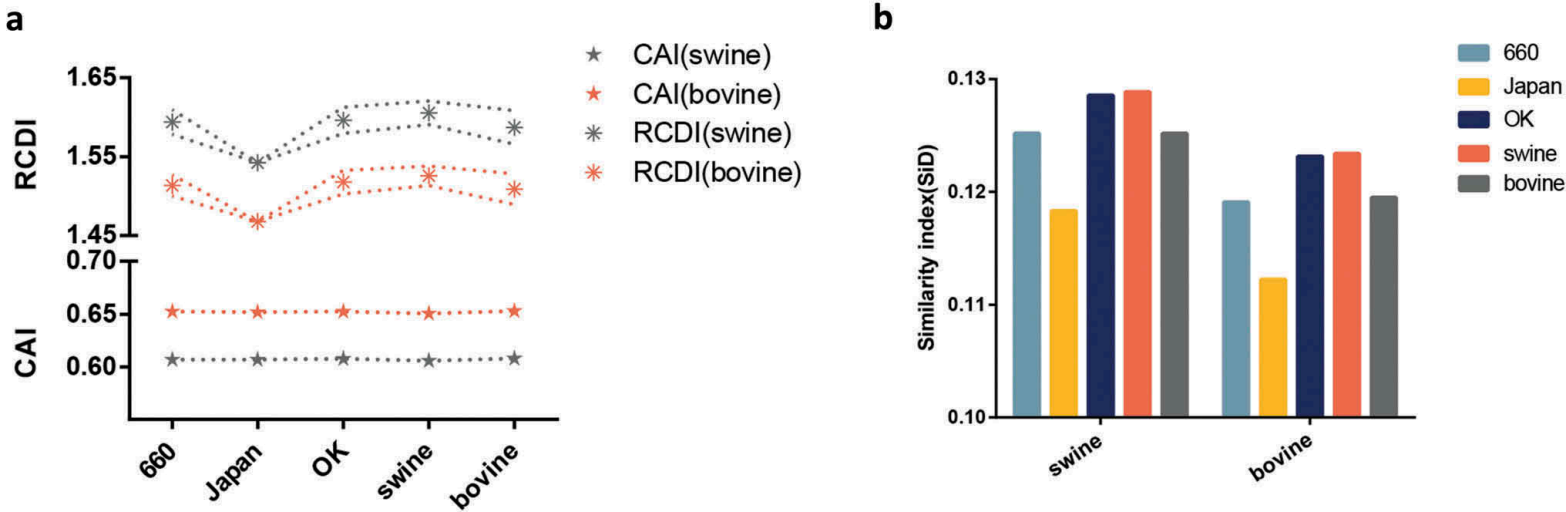
(a) CAI analysis (bottom panel represented by a symbol star) and RCDI analysis (upper panel represented by a symbol asterisk) of the HEF gene in relation to the natural hosts. The lines in the RCDI analysis in each host represent the upper and lower limit. (b) SiD analysis of the IDV HEF gene. The D/660, D/Japan, and D/OK clades are represented in light blue, yellow, dark blue. The swine, goat, and bovine hosts are represented in grey, dark green, and orange, respectively. The x axis represents the sequences belonging to different clades or identified in different hosts.

Next, we explored the three currently identified hosts (Bos taurus, Sus scrofa, and Capra hircus) against the three clades. The CAI represents the expression level of a gene based on its codon usage pattern. As shown in Figure 5a, the highest CAI value of all IDV strains taken together was for bovine (0.653 ± 0.003), followed closely by goat (0.647 ± 0.003) and then swine (0.607 ± 0.003). In particular, the D/660 clade had the highest CAI value (0.6527 ± 0.00378 for bovine, 0.6477 ± 0.004 for goat and 0.607 ± 0.004 for swine) compared to the other two clades, followed by the D/OK and the D/Japan clades. We also performed RCDI analysis to understand the deoptimization of all strains in relation to the individual clades. The RCDI values of all strains to swine were higher than to bovine and goat. In addition, the clades with the highest and lowest RCDI values in relation to swine were the D/OK (1.594 ± 0.0154) and D/Japan (1.5423 ± 0.0045) clades, respectively. The same trend was found for bovine and goat (Figure 5a). The results between different hosts in individual clade and host were significant, with a p value less than 0.01, in relation to both CAI and RCDI.

For the PB1 gene, the lowest CAI value was observed in swine while the RCDI value was the highest for swine (Fig S1D)

### High selection pressure in swine influences the IDV codon usage pattern

To understand how the codon usage patterns of the three hosts affect the virus codon usage pattern, we performed SiD analysis (Figure 5b). We found that in the HEF gene, the SiD value of swine (0.126) was higher than bovine (0.120) and goat (0.117) indicating that during IDV evolution, swine had a greater impact on the virus than bovine and goat. Moreover, the D/OK clade displayed the highest value, followed by the D/660 and the D/Japan clades. Similar results observed for the PB1 gene (Fig S1E).

## Discussion

IDV was first discovered in the United States, but is present in China, Japan, France, Ireland, and Italy [4–8]. It displays a wide range of host, in particular infecting swine and bovine, but with serological evidence in small ruminants, ferrets, and humans [2,3]. A recent study explored the codon usage pattern reflecting the evolutionary changes of IDV to survive and adapt to a multi-host environment based on analysis of the HEF and PB1 genes. The phylogeny based on the HEF gene revealed the existence of two clades: D/OK and D/660 [23]. A later study showed that the strains detected in Japan clustered apart from others in the phylogeny [41], which was confirmed in our study. Given the high degree of conservation of the PB1, its evolution in respect to codon usage pattern was also studied here.

Nucleotides A/U more frequently used and the most common at the third position of synonymous codons in both the HEF and PB1 genes. Additionally, the usage bias towards A- and U-ended codons was also revealed in RSCU analysis. We found an ENC value higher than 35, indicative of a low degree of preference. Other studies have also reported a low IAV codon usage bias, including the CIV H3N2 HA gene (ENC = 53.22 ± 0.316), the AIV H1N1pdm (52.50) [42], and EIV H3N8 (52.09) [21], which might allow the virus to replicate in the host environment by reducing competition [12]. Therefore, we hypothesized that the lower codon usage pattern in IDV could aid in proliferation and facilitate infectivity in multiple hosts. Additionally, the D/Japan clade of the HEF gene displayed the lowest codon usage bias. However, this result might be biased given the limited number of sequences from Japan. Moreover, the strains isolated from swine had a higher codon usage bias compared to bovine. This might be further confirmed by CAI analysis that indicated that IDV is more adapted to bovine than swine. Both in eukaryotes and prokaryotes, the codon usage is mainly influenced by the balance of mutation pressure and natural selection [43,44]. Here, we found by ENC-plot and PR2 analyses that IDV is influenced by mutation pressure and natural selection with variable degrees. Generally, it is considered that the G/C or A/U abundance relate to the corresponding RSCU pattern. Thus, the tendency of mutation pressure can be verified by the preferred ended codons [12]. Furthermore, using neutrality analysis we demonstrated that natural selection controls the wide IDV host range, confirmed by a slope of regression line less than 1. This might due to the weak codon usage bias in IDV was caused by natural selection when the viruses try to adapted to the host cells [45].

We also analysis if the codon usage pattern relates to the specific hosts. We found that the CAI values of bovine and goat were higher than swine, in agreement with RCDI analysis [46]. This phenomenon was observed for both the HEF and the PB1 genes. This might lead to a lower IDV protein synthesis in swine compared to other hosts [33]. In addition, in order to ensure accurate replication, survival, and efficient pathogenicity in multi-hosts, the virus must balance between complex codon adaption and deoptimization [12]. Interestingly, the SiD value of swine was higher than that of bovine and goat, indicating that the selection pressure of swine on IDV was greater than bovine and goat, in agreement with neutrality analysis, especially for the D/OK clade. We therefore hypothesize a strong link between IDV and swine, although bovine was always suggested as the primary IDV host [2,23]. In summary, the potential IDV natural hosts could be either swine, bovine [12], or both. Thus, the threat of IDV to public health should be more carefully monitored.

In conclusion, here we analyzed the overall codon usage pattern of the IDV HEF and PB1 genes to better understand the evolutionary changes of this novel influenza virus. This study aids into the prevention of widespread IDV, although its origin and natural ecology remain unknown.
