# Supplementary material for: Evolutionary changes of the novel Influenza D virus hemagglutinin-esterase fusion gene revealed by the codon usage pattern
Source: Virulence. 2018 Nov 26;10(1):1–9. doi: 10.1080/21505594.2018.1551708 (PMC6298762; doi:10.1080/21505594.2018.1551708)
Supplement: Supplemental Material [file kvir-10-01-1551708-s001.docx]

Table S1 Strain information and the composition of the codon parameters.

**HEF gene:**

| Strain name | GC_1s_ | GC_2s_ | GC_12s_ | GC_3s_ | A% | C% | G% | U% | AU% | GC% | U_3s_ | C_3s_ | A_3s_ | G3s | ENC | Gravy | Aromo | Axis1 | Axis2 |
| --- | --- | --- | --- | --- | --- | --- | --- | --- | --- | --- | --- | --- | --- | --- | --- | --- | --- | --- | --- |
| KF425655_D/bovine/Minnesota/628/2013 | 0.471 | 0.468 | 0.470 | 0.366 | 31.160 | 19.590 | 23.930 | 25.270 | 56.430 | 43.520 | 0.338 | 0.234 | 0.444 | 0.234 | 48.610 | -0.158 | 0.089 | 0.009 | 0.014 |
| KM392485_D/bovine/Texas/3-13/2011 | 0.471 | 0.470 | 0.471 | 0.363 | 31.160 | 19.480 | 23.980 | 25.320 | 56.480 | 43.460 | 0.342 | 0.232 | 0.442 | 0.230 | 48.020 | -0.140 | 0.089 | -0.058 | 0.056 |
| KM392499_D/bovine/Kansas/14-22/2012 | 0.470 | 0.464 | 0.467 | 0.366 | 31.520 | 19.530 | 23.770 | 25.120 | 56.640 | 43.300 | 0.337 | 0.232 | 0.447 | 0.237 | 48.720 | -0.161 | 0.089 | 0.021 | -0.004 |
| KX768841_D/swine/Italy/354017/2015 | 0.468 | 0.461 | 0.464 | 0.364 | 31.630 | 19.530 | 23.510 | 25.120 | 56.750 | 43.040 | 0.333 | 0.241 | 0.453 | 0.228 | 48.210 | -0.156 | 0.089 | 0.010 | 0.022 |
| KF425662_D/bovine/Oklahoma/660/2013 | 0.472 | 0.471 | 0.471 | 0.353 | 31.310 | 19.310 | 23.860 | 25.470 | 56.780 | 43.170 | 0.350 | 0.227 | 0.448 | 0.222 | 48.300 | -0.164 | 0.089 | -0.061 | 0.066 |
| KX768820_D/swine/Italy/268344-2/2015 | 0.466 | 0.460 | 0.463 | 0.361 | 31.730 | 19.300 | 23.580 | 25.340 | 57.070 | 42.880 | 0.335 | 0.234 | 0.458 | 0.228 | 48.150 | -0.161 | 0.090 | 0.011 | 0.021 |
| KM392471_D/bovine/Nebraska/9-5/2012 | 0.473 | 0.470 | 0.471 | 0.357 | 31.210 | 19.430 | 23.880 | 25.320 | 56.530 | 43.310 | 0.345 | 0.231 | 0.449 | 0.222 | 48.400 | -0.154 | 0.089 | -0.053 | 0.057 |
| KM392506_D/bovine/Kansas/11-8/2012 | 0.475 | 0.471 | 0.473 | 0.357 | 31.260 | 19.460 | 23.960 | 25.260 | 56.520 | 43.420 | 0.341 | 0.231 | 0.452 | 0.224 | 48.030 | -0.149 | 0.089 | -0.042 | 0.048 |
| KM392478_D/bovine/Kansas/1-35/2010 | 0.470 | 0.465 | 0.467 | 0.366 | 31.420 | 19.480 | 23.880 | 25.170 | 56.590 | 43.360 | 0.335 | 0.234 | 0.449 | 0.234 | 48.120 | -0.159 | 0.089 | 0.023 | -0.005 |
| Influenza_D_virus_D/swine/Guangdong/NN13/2017 | 0.471 | 0.464 | 0.467 | 0.370 | 31.370 | 19.620 | 23.860 | 25.160 | 56.530 | 43.480 | 0.333 | 0.236 | 0.447 | 0.238 | 47.380 | -0.148 | 0.089 | 0.064 | -0.051 |
| Influenza_D_virus_D/swine/Guangdong/LX-2/2018 | 0.471 | 0.465 | 0.468 | 0.370 | 31.420 | 19.700 | 23.850 | 25.040 | 56.460 | 43.550 | 0.332 | 0.238 | 0.448 | 0.236 | 47.530 | -0.154 | 0.089 | 0.063 | -0.052 |
| Influenza_D_virus_D/bovine/Guangdong/SK/2018 | 0.469 | 0.464 | 0.467 | 0.373 | 31.370 | 19.720 | 23.810 | 25.100 | 56.470 | 43.530 | 0.332 | 0.240 | 0.445 | 0.238 | 47.600 | -0.152 | 0.089 | 0.063 | -0.050 |
| Influenza_D_virus_D/bovine/Guangdong/SQ/2018 | 0.469 | 0.464 | 0.467 | 0.371 | 31.520 | 19.670 | 23.810 | 25.000 | 56.520 | 43.480 | 0.330 | 0.238 | 0.449 | 0.238 | 47.280 | -0.163 | 0.089 | 0.061 | -0.052 |
| Influenza_D_virus_D/bovine/Guangdong/25969/2017 | 0.467 | 0.464 | 0.466 | 0.370 | 31.470 | 19.620 | 23.760 | 25.160 | 56.630 | 43.380 | 0.333 | 0.236 | 0.447 | 0.238 | 47.330 | -0.151 | 0.089 | 0.064 | -0.051 |
| Influenza_D_virus_D/bovine/Guangdong/YC/2017 | 0.471 | 0.464 | 0.467 | 0.370 | 31.370 | 19.670 | 23.810 | 25.160 | 56.530 | 43.480 | 0.333 | 0.236 | 0.447 | 0.238 | 46.970 | -0.151 | 0.089 | 0.065 | -0.052 |
| LC270268_D/bovine/Miyazaki/B22/2016 | 0.478 | 0.466 | 0.472 | 0.374 | 30.950 | 20.030 | 23.910 | 25.100 | 56.050 | 43.940 | 0.340 | 0.244 | 0.434 | 0.234 | 49.200 | -0.143 | 0.092 | 0.016 | -0.040 |
| JQ922308_D/swine/Oklahoma/1334/2011 | 0.471 | 0.464 | 0.467 | 0.367 | 31.420 | 19.480 | 23.930 | 25.170 | 56.590 | 43.410 | 0.337 | 0.234 | 0.446 | 0.236 | 48.440 | -0.159 | 0.088 | 0.025 | 0.000 |
| LC318668_D/bovine/Yamagata/10710/2016 | 0.480 | 0.464 | 0.472 | 0.376 | 30.950 | 20.030 | 23.960 | 25.050 | 56.000 | 43.990 | 0.338 | 0.244 | 0.434 | 0.236 | 49.140 | -0.145 | 0.090 | 0.015 | -0.039 |
| KX768834_D/swine/Italy/254578/2015 | 0.468 | 0.461 | 0.464 | 0.358 | 31.780 | 19.330 | 23.570 | 25.320 | 57.100 | 42.900 | 0.335 | 0.234 | 0.462 | 0.224 | 48.000 | -0.157 | 0.090 | 0.011 | 0.021 |
| KX768827_D/swine/Italy/173287-4/2016 | 0.468 | 0.456 | 0.462 | 0.361 | 31.730 | 19.330 | 23.510 | 25.430 | 57.160 | 42.840 | 0.335 | 0.236 | 0.457 | 0.225 | 47.930 | -0.147 | 0.088 | 0.020 | 0.021 |
| LC128433_D/bovine/Ibaraki/7768/2016 | 0.480 | 0.464 | 0.472 | 0.374 | 30.950 | 20.030 | 23.910 | 25.100 | 56.050 | 43.940 | 0.340 | 0.244 | 0.434 | 0.234 | 49.010 | -0.145 | 0.090 | 0.012 | -0.038 |
| KU171129_D/bovine/Mexico/S62/2015 | 0.471 | 0.464 | 0.467 | 0.367 | 31.420 | 19.480 | 23.930 | 25.170 | 56.590 | 43.410 | 0.337 | 0.234 | 0.446 | 0.236 | 48.440 | -0.159 | 0.088 | 0.025 | 0.000 |
| KU171128_D/bovine/Mexico/S56/2015 | 0.471 | 0.468 | 0.470 | 0.361 | 31.370 | 19.690 | 23.670 | 25.270 | 56.640 | 43.360 | 0.340 | 0.239 | 0.448 | 0.219 | 48.460 | -0.157 | 0.088 | -0.075 | 0.073 |
| KU171127_D/bovine/Mexico/S8/2015 | 0.470 | 0.468 | 0.469 | 0.364 | 31.160 | 19.590 | 23.820 | 25.430 | 56.590 | 43.410 | 0.343 | 0.237 | 0.440 | 0.226 | 48.380 | -0.151 | 0.088 | -0.065 | 0.065 |
| KU171126_D/bovine/Mexico/S7/2015 | 0.470 | 0.468 | 0.469 | 0.364 | 31.160 | 19.590 | 23.820 | 25.430 | 56.590 | 43.410 | 0.343 | 0.237 | 0.440 | 0.226 | 48.380 | -0.151 | 0.088 | -0.065 | 0.065 |
| KT592522_D/bovine/Italy/1/2014 | 0.471 | 0.461 | 0.466 | 0.367 | 31.520 | 19.530 | 23.770 | 25.170 | 56.690 | 43.300 | 0.329 | 0.241 | 0.455 | 0.228 | 48.090 | -0.145 | 0.088 | 0.016 | 0.012 |
| KT581418_D/bovine/Mississippi/C00030P/2014 | 0.467 | 0.464 | 0.465 | 0.369 | 31.580 | 19.590 | 23.720 | 25.120 | 56.700 | 43.310 | 0.333 | 0.237 | 0.449 | 0.234 | 48.320 | -0.171 | 0.088 | 0.027 | 0.003 |
| KT581417_D/bovine/Mississippi/C00014N/2014 | 0.470 | 0.470 | 0.470 | 0.350 | 31.580 | 19.380 | 23.620 | 25.430 | 57.010 | 43.000 | 0.347 | 0.228 | 0.455 | 0.218 | 48.210 | -0.158 | 0.088 | -0.062 | 0.082 |
| KT581416_D/bovine/Mississippi/C00013N/2014 | 0.470 | 0.470 | 0.470 | 0.350 | 31.580 | 19.380 | 23.620 | 25.430 | 57.010 | 43.000 | 0.347 | 0.228 | 0.455 | 0.218 | 48.210 | -0.158 | 0.088 | -0.062 | 0.082 |
| KT581412_D/bovine/Mississippi/C00046N/2014 | 0.470 | 0.464 | 0.467 | 0.367 | 31.520 | 19.590 | 23.770 | 25.120 | 56.640 | 43.360 | 0.333 | 0.236 | 0.451 | 0.234 | 48.390 | -0.171 | 0.087 | 0.022 | 0.011 |
| KM015494_D/bovine/Shandong/Y125/2014 | 0.469 | 0.463 | 0.466 | 0.368 | 31.420 | 19.510 | 23.810 | 25.260 | 56.680 | 43.320 | 0.335 | 0.234 | 0.447 | 0.238 | 47.380 | -0.140 | 0.089 | 0.059 | -0.043 |
| KM015501_D/bovine/Shandong/Y127/2014 | 0.467 | 0.464 | 0.466 | 0.368 | 31.470 | 19.510 | 23.810 | 25.210 | 56.680 | 43.320 | 0.335 | 0.234 | 0.446 | 0.237 | 47.510 | -0.148 | 0.089 | 0.058 | -0.042 |
| KM015508_D/bovine/Shandong/Y217/2014 | 0.467 | 0.464 | 0.466 | 0.370 | 31.420 | 19.620 | 23.760 | 25.210 | 56.630 | 43.380 | 0.335 | 0.236 | 0.445 | 0.238 | 47.500 | -0.152 | 0.089 | 0.063 | -0.049 |
| KM392492_D/bovine/Kansas/13-21/2012 | 0.474 | 0.471 | 0.473 | 0.361 | 31.010 | 19.530 | 24.030 | 25.430 | 56.440 | 43.560 | 0.344 | 0.231 | 0.442 | 0.228 | 48.570 | -0.153 | 0.088 | -0.321 | -0.197 |
| KT592526_D/bovine/Italy/46484/2015 | 0.470 | 0.462 | 0.466 | 0.363 | 31.630 | 19.430 | 23.720 | 25.220 | 56.850 | 43.150 | 0.332 | 0.237 | 0.457 | 0.225 | 47.230 | -0.153 | 0.088 | 0.023 | 0.016 |
| KT592533_D/swine/Italy/199724-3/2015 | 0.468 | 0.461 | 0.464 | 0.364 | 31.680 | 19.450 | 23.630 | 25.230 | 56.910 | 43.080 | 0.332 | 0.237 | 0.456 | 0.227 | 47.830 | -0.150 | 0.088 | 0.011 | 0.017 |
| KF425669_D/bovine/Minnesota/729/2013 | 0.471 | 0.468 | 0.470 | 0.366 | 31.210 | 19.590 | 23.930 | 25.270 | 56.480 | 43.520 | 0.339 | 0.233 | 0.444 | 0.234 | 48.630 | -0.163 | 0.088 | 0.008 | 0.015 |

**PB1 gene:**

| Strain name | GC1S | GC2S | GC12S | GC3S | A% | C% | G% | U% | AU% | GC% | U3s | C3s | A3s | G3s | ENC | Gravy | Aromo | Axis1 | Axis2 |
| --- | --- | --- | --- | --- | --- | --- | --- | --- | --- | --- | --- | --- | --- | --- | --- | --- | --- | --- | --- |
| JQ922306_D/swine/Oklahoma/1334/2011 | 0.431 | 0.399 | 0.415 | 0.389 | 37.100 | 18.930 | 21.700 | 22.270 | 59.370 | 40.630 | 0.274 | 0.267 | 0.503 | 0.250 | 49.600 | -0.520 | 0.084 | 0.011 | 0.016 |
| KF425653_D/bovine/Minnesota/628/2013 | 0.431 | 0.401 | 0.416 | 0.392 | 37.150 | 19.220 | 21.550 | 22.080 | 59.230 | 40.770 | 0.266 | 0.275 | 0.506 | 0.244 | 48.900 | -0.522 | 0.084 | 0.024 | 0.004 |
| KF425660_D/bovine/Oklahoma/660/2013 | 0.429 | 0.398 | 0.413 | 0.385 | 37.100 | 18.740 | 21.650 | 22.510 | 59.610 | 40.390 | 0.281 | 0.261 | 0.501 | 0.250 | 48.470 | -0.517 | 0.084 | 0.016 | -0.006 |
| KF425667_D/bovine/Minnesota/729/2013 | 0.431 | 0.401 | 0.416 | 0.392 | 37.150 | 19.220 | 21.550 | 22.080 | 59.230 | 40.770 | 0.266 | 0.275 | 0.506 | 0.244 | 48.900 | -0.522 | 0.084 | 0.024 | 0.004 |
| KM015492 _D/bovine/Shandong/Y125/2014 | 0.431 | 0.399 | 0.415 | 0.383 | 36.950 | 18.710 | 21.710 | 22.620 | 59.570 | 40.420 | 0.281 | 0.260 | 0.503 | 0.247 | 48.980 | -0.513 | 0.084 | 0.009 | -0.072 |
| KM015499_D/bovine/Shandong/Y127/2014 | 0.433 | 0.400 | 0.416 | 0.383 | 36.860 | 18.710 | 21.810 | 22.620 | 59.480 | 40.520 | 0.283 | 0.258 | 0.500 | 0.249 | 49.050 | -0.512 | 0.083 | 0.009 | -0.076 |
| KM015506_D/bovine/Shandong/Y217/2014 | 0.434 | 0.399 | 0.416 | 0.386 | 36.860 | 18.810 | 21.810 | 22.520 | 59.380 | 40.620 | 0.280 | 0.262 | 0.501 | 0.249 | 49.200 | -0.507 | 0.084 | 0.010 | -0.068 |
| KM392469_D/bovine/Nebraska/9-5/2012 | 0.429 | 0.398 | 0.413 | 0.395 | 37.200 | 19.170 | 21.550 | 22.080 | 59.280 | 40.720 | 0.267 | 0.276 | 0.503 | 0.248 | 49.210 | -0.520 | 0.084 | 0.017 | 0.009 |
| KM392476_D/bovine/Kansas/1-35/2010 | 0.431 | 0.401 | 0.416 | 0.395 | 37.050 | 19.120 | 21.750 | 22.080 | 59.130 | 40.870 | 0.266 | 0.273 | 0.503 | 0.250 | 49.480 | -0.521 | 0.084 | 0.023 | 0.012 |
| KM392483_D/bovine/Texas/3-13/2011 | 0.428 | 0.401 | 0.414 | 0.385 | 37.200 | 18.930 | 21.510 | 22.320 | 59.520 | 40.440 | 0.276 | 0.267 | 0.506 | 0.244 | 48.830 | -0.524 | 0.085 | 0.021 | 0.012 |
| KM392490_D/bovine/Kansas/13-21/2012 | 0.432 | 0.401 | 0.416 | 0.395 | 36.960 | 19.120 | 21.790 | 22.130 | 59.090 | 40.910 | 0.270 | 0.272 | 0.499 | 0.252 | 48.910 | -0.521 | 0.084 | 0.023 | 0.056 |
| KM392497_D/bovine/Kansas/14-22/2012 | 0.431 | 0.398 | 0.414 | 0.385 | 37.200 | 18.790 | 21.650 | 22.370 | 59.570 | 40.440 | 0.278 | 0.262 | 0.505 | 0.250 | 49.720 | -0.519 | 0.084 | 0.006 | 0.009 |
| KM392504_D/bovine/Kansas/11-8/2012 | 0.429 | 0.399 | 0.414 | 0.388 | 37.150 | 18.880 | 21.650 | 22.320 | 59.470 | 40.530 | 0.274 | 0.267 | 0.504 | 0.248 | 48.890 | -0.519 | 0.084 | 0.010 | -0.001 |
| KT581410_D/bovine/Mississippi/C00046N/2014 | 0.431 | 0.399 | 0.415 | 0.388 | 37.150 | 18.880 | 21.700 | 22.270 | 59.420 | 40.580 | 0.274 | 0.265 | 0.505 | 0.250 | 49.560 | -0.520 | 0.084 | 0.020 | 0.003 |
| KT592521 _D/bovine/Italy/1/2014 | 0.431 | 0.399 | 0.415 | 0.401 | 36.810 | 19.120 | 21.890 | 22.170 | 58.980 | 41.010 | 0.270 | 0.274 | 0.492 | 0.258 | 49.260 | -0.520 | 0.084 | 0.033 | 0.044 |
| KT592524 _D/bovine/Italy/46484/2015 | 0.429 | 0.399 | 0.414 | 0.398 | 36.860 | 19.030 | 21.840 | 22.270 | 59.130 | 40.870 | 0.272 | 0.272 | 0.494 | 0.256 | 49.360 | -0.520 | 0.084 | 0.023 | 0.025 |
| KT592531 _D/swine/Italy/199724-3/2015 | 0.431 | 0.400 | 0.415 | 0.400 | 36.720 | 19.050 | 21.970 | 22.250 | 58.970 | 41.020 | 0.274 | 0.271 | 0.489 | 0.260 | 49.680 | -0.527 | 0.085 | 0.035 | 0.043 |
| KU710434_D/bovine/Mexico/S7/2015 | 0.435 | 0.399 | 0.417 | 0.388 | 37.240 | 19.270 | 21.460 | 22.030 | 59.270 | 40.730 | 0.269 | 0.276 | 0.510 | 0.238 | 49.020 | -0.529 | 0.084 | 0.022 | 0.008 |
| KU710435_D/bovine/Mexico/S8/2015 | 0.435 | 0.399 | 0.417 | 0.388 | 37.240 | 19.270 | 21.460 | 22.030 | 59.270 | 40.730 | 0.269 | 0.276 | 0.510 | 0.238 | 49.020 | -0.529 | 0.084 | 0.022 | 0.008 |
| KU710436_D/bovine/Mexico/S56/2015 | 0.426 | 0.401 | 0.413 | 0.386 | 37.200 | 18.930 | 21.510 | 22.370 | 59.570 | 40.440 | 0.272 | 0.272 | 0.508 | 0.240 | 48.820 | -0.521 | 0.084 | 0.023 | -0.061 |
| KU710437_D/bovine/Mexico/S62/2015 | 0.429 | 0.403 | 0.416 | 0.390 | 36.610 | 19.000 | 21.730 | 22.670 | 59.280 | 40.730 | 0.276 | 0.271 | 0.499 | 0.245 | 49.200 | -0.489 | 0.086 | 0.028 | -0.058 |
| KX768825_D/swine/Italy/173287-4/2016 | 0.431 | 0.399 | 0.415 | 0.396 | 36.810 | 18.980 | 21.890 | 22.320 | 59.130 | 40.870 | 0.276 | 0.269 | 0.492 | 0.258 | 49.440 | -0.520 | 0.084 | 0.034 | 0.041 |
| KX768832_D/swine/Italy/254578/2015 | 0.433 | 0.400 | 0.416 | 0.397 | 36.710 | 19.000 | 22.000 | 22.290 | 59.000 | 41.000 | 0.273 | 0.269 | 0.493 | 0.257 | 49.510 | -0.515 | 0.084 | 0.027 | 0.027 |
| KX768839_D/swine/Italy/354017/2015 | 0.431 | 0.399 | 0.415 | 0.399 | 36.810 | 19.070 | 21.890 | 22.220 | 59.030 | 40.960 | 0.272 | 0.272 | 0.492 | 0.258 | 49.400 | -0.519 | 0.084 | 0.029 | 0.036 |
| LC128438 _D/bovine/Ibaraki/7768/2016 | 0.430 | 0.403 | 0.417 | 0.386 | 36.490 | 18.600 | 22.050 | 22.860 | 59.350 | 40.650 | 0.293 | 0.252 | 0.487 | 0.261 | 51.090 | -0.534 | 0.086 | -0.120 | -0.029 |
| LC270266_D/bovine/Miyazaki/B22/2016 | 0.428 | 0.403 | 0.415 | 0.380 | 36.680 | 18.460 | 21.900 | 22.960 | 59.640 | 40.360 | 0.296 | 0.251 | 0.492 | 0.254 | 50.880 | -0.535 | 0.086 | -0.119 | -0.030 |
| LC318666 _D/bovine/Yamagata/10710/2016 | 0.429 | 0.403 | 0.416 | 0.385 | 36.590 | 18.560 | 22.000 | 22.860 | 59.450 | 40.560 | 0.294 | 0.251 | 0.488 | 0.260 | 51.400 | -0.544 | 0.086 | -0.259 | 0.044 |

Table S2. The RSCU value of 59 codons encoding 18 amino acids. The optimal codons are shown in bold.

| **Codon** | **RSCU** | **Codon** | **RSCU** |
| --- | --- | --- | --- |
| **GCA(A)** | **1.56** | CCA(P) | 1.47 |
| GCC(A) | 1.01 | CCC(P) | 0.52 |
| GCG(A) | 0.19 | CCG(P) | 0.31 |
| GCT(A) | 1.24 | **CCT(P)** | **1.69** |
| **TGC(C)** | **1.21** | **CAA(Q)** | **1.05** |
| TGT(C) | 0.79 | CAG(Q) | 0.95 |
| **GAC(D)** | **1.07** | **AGA(R)** | **2.79** |
| GAT(D) | 0.93 | AGG(R) | 2.23 |
| **GAA(E)** | **1.40** | CGA(R) | 0.70 |
| GAG(E) | 0.60 | CGC(R) | 0.00 |
| **TTC(F)** | **1.22** | CGG(R) | 0.13 |
| TTT(F) | 0.78 | CGT(R) | 0.14 |
| **GGA(G)** | **2.41** | AGC(S) | 0.87 |
| GGC(G) | 0.09 | AGT(S) | 0.75 |
| GGG(G) | 1.31 | **TCA(S)** | **2.02** |
| GGT(G) | 0.19 | TCC(S) | 0.63 |
| CAC(H) | 0.87 | TCG(S) | 0.60 |
| **CAT(H)** | **1.13** | TCT(S) | 1.13 |
| **ATA(I)** | **1.63** | **ACA(T)** | **1.83** |
| ATC(I) | 0.68 | ACC(T) | 0.72 |
| ATT(I) | 0.69 | ACG(T) | 0.27 |
| **AAA(K)** | **1.42** | ACT(T) | 1.18 |
| AAG(K) | 0.58 | GTA(V) | 0.82 |
| CTA(L) | 1.05 | GTC(V) | 0.69 |
| CTC(L) | 0.53 | GTG(V) | 0.89 |
| CTG(L) | 0.83 | **GTT(V)** | **1.59** |
| CTT(L) | 0.58 | **TAC(Y)** | **1.42** |
| TTA(L) | 1.05 | TAT(Y) | 0.58 |
| **TTG(L)** | **1.96** |  |  |
| **AAC(N)** | **1.10** |  |  |
| AAT(N) | 0.90 |  |  |
|  |  |  |  |
